# Experience and confidence in health technologies: evidence from malaria testing and treatment in Western Kenya

**DOI:** 10.1186/s12889-022-14102-y

**Published:** 2022-09-06

**Authors:** Judith N. Mangeni, Lucy Abel, Steve M. Taylor, Andrew Obala, Wendy Prudhomme O’Meara, Indrani Saran

**Affiliations:** 1 School of Public Health, College of Health Sciences, Moi University, P.O BOX 512-30100, Eldoret, Kenya; 2 Academic Model Providing Access to Healthcare, Moi Teaching and Referral Hospital, Eldoret, Kenya; 3 Division of Infectious Diseases, School of Medicine, Duke University, Durham, NC USA; 4 School of Medicine, College of Health Sciences, Moi University, Eldoret, Kenya; 5 Duke Global Health Institute, Duke University, Durham, NC USA; 6 Boston College of Social Work, McGuinn Hall 305, Newton, MA USA

**Keywords:** Experience, Confidence, Health technologies, Malaria testing, Western Kenya

## Abstract

**Background:**

Low adoption of effective health technologies increases illness morbidity and mortality worldwide. In the case of malaria, effective tools such as malaria rapid diagnostic tests (RDTs) and artemisinin-combination therapies (ACTs) are both under-used and used inappropriately. Individuals’ confidence in RDTs and ACTs likely affects the uptake of these tools.

**Methods:**

In a cohort of 36 households (280 individuals) in Western Kenya observed for 30 months starting in June 2017, we examined if experience with RDTs and ACTs changes people’s beliefs about these technologies and how those beliefs affect treatment behavior. Household members requested a free RDT from the study team any time they suspected a malaria illness, and positive RDT results were treated with a free ACT. We conducted annual, monthly, and sick visit surveys to elicit beliefs about the accuracy of malaria RDT results and the effectiveness of ACTs. Beliefs were elicited on a 5-point Likert scale from “very unlikely” to “very likely.”

**Results:**

Over the study period, the percentage of survey respondents that said a hypothetical negative RDT result was “very likely” to be correct increased from approximately 55% to 75%. Controlling for initial beliefs, people who had been tested at least once with an RDT in the past year had 3.6 times higher odds (95% CI [1 1.718 7.679], *P* = 0.001) of saying a negative RDT was “very likely” to be correct. Confidence in testing was associated with treatment behavior: those who believed a negative RDT was “very likely” to be correct had 1.78 times higher odds (95% CI [1.079 2.934], *P* = 0.024) of adhering to a negative RDT result (by not taking ACTs) than those who were less certain about the accuracy of negative RDTs. Adherence to a negative test also affected subsequent beliefs: controlling for prior beliefs, those who had adhered to their previous test result had approximately twice the odds (OR = 2.19, 95% CI [1.661 2.904], *P* < 0.001) of saying that a hypothetical negative RDT was “very likely” to be correct compared to those who had not adhered.

**Conclusions:**

Our results suggest that greater experience with RDTs can not only increase people’s confidence in their accuracy but also improve adherence to the test result.

**Supplementary Information:**

The online version contains supplementary material available at 10.1186/s12889-022-14102-y.

## Background

In both high and low-income countries, underuse of effective health technologies, such as vaccinations and insecticide-treated bed nets, leads to high levels of avertable morbidity and mortality [1]. However, little is still known about how people’s beliefs evolve as they learn, and incorporate new information, about health technologies. There is some evidence that initial subsidies can encourage future adoption, while other research suggests that people can learn about the value of new technologies from their social networks [2–6].

In the case of malaria, there exist two very effective and accessible health tools that have contributed substantially to reductions in malaria burden: rapid diagnostic tests (RDTs) for malaria diagnosis and artemisinin-combination therapies (ACTs) for malaria treatment. Despite the availability of these tools, however, malaria remains a leading public health problem globally and especially in Sub-Saharan Africa. In 2020, approximately 241 million malaria cases were recorded globally and malaria killed an estimated 627,000 people [7].

Prompt diagnosis and appropriate treatment is key to preventing these unnecessary deaths. In 2010, the WHO recommended that all patients suspected of malaria be tested using either microscopy or RDT before receiving anti-malaria treatment [8]. In the last decade, there has been a massive scale up of RDT supply to Sub-Saharan Africa with the number of manufacturer-reported global RDTs sales increasing from less than 100 million in 2010 to approximately 419 million in 2020 [7]. This has made the WHO goal of “Test and Treat” much more feasible especially in resource-limited settings. RDTs are accurate, require minimal labor and training, and give results in approximately 15 min [9–13].

Despite these advantages of RDTs, presumptive treatment of fevers with anti-malarials has continued by both clinicians and patients, many of whom still believe all febrile illnesses are malaria [14–16]. Treating those without malaria with ACTs leads to delays in appropriate management of the illness, wastage of valuable drugs and potentially increases the spread of drug resistance [17–20]. Although Kenya adopted the WHO guidelines on “Test and Treat” a decade ago, studies in western Kenya indicate that presumptive malaria treatment, and lack of adherence to negative RDT results are common practices, especially in areas of high transmission [21–23].

At the same time, there is evidence that many children with malaria do not receive timely treatment with ACTs, drugs that are very effective in reducing morbidity and mortality from the disease [24–27]. Individuals’ under-estimation about the effectiveness of ACTs relative to older anti-malarial drugs could hamper uptake of the drug. A better understanding of people’s beliefs about health technologies such as RDTs and ACTs, including how these beliefs change over time, and how they relate to treatment behaviors, could improve design of interventions to encourage uptake and appropriate use [28].

We followed a cohort of households in a high transmission malaria region in Western Kenya for 30 months. During this period, household members could request a free RDT from the study team for any suspected malaria illness. Individuals with RDT-positive infections were offered free treatment with an ACT. We investigated if individuals’ beliefs about RDTs affected their testing and treatment decisions, and how those decisions subsequently influenced their beliefs about these key health technologies. The main objective of this study was to determine if experience with RDTs increases confidence in RDTs, and whether confidence in RDTs in turn improves adherence to the test result. To the best of our knowledge, this is the first longitudinal study that has investigated malaria beliefs over an extended period.

## Methods

### Study setting and design

This study was part of a larger study that was designed to better understand the spatial scales of malaria transmission events in the Webuye Health and Demographic Surveillance System (HDSS). The Webuye HDSS was established in 2007 to provide reliable demographic, health and economic information for planning as well as provide a platform for health research [29]. The main study (our study was a sub-analysis) enrolled a subset of households into a longitudinal cohort using an open cohort design. “We enrolled an index household at random and then neighboring households radiating outwards until 12 households were enrolled per village in a natural grouping”[30]. The three villages enrolled into the study had higher than the average transmission in the area (malaria hotspots). All members of the household who were older than 12 months and regularly slept in the household were enrolled into the study. This resulted in a total sample size of 36 households and 280 participants who were followed between June 2017 and December 2019. Two households were replaced with neighboring households when the entire household migrated (the two migrating households are not included in this analysis). The three villages were located within two sub counties (Webuye East and Webuye West Sub-Counties) in western Kenya. This region has had high transmission of malaria throughout the year with small seasonal variations [31]. The study setting is described in detail elsewhere [32–34].

### Study procedures

We conducted three types of surveys with households. The first type of survey was an “annual survey” conducted at baseline and repeated a year later. All members of these households including children above one year were included in the enrollment. The household respondent was either the male or female who heads the household and were generally a parent or primary caregiver of children under 18. We collected demographic information on household members, as well as details about housing characteristics, household assets and bed net use from a designated household respondent (usually the male or female household head). All adults aged 18 and above were also asked about their beliefs about malaria testing and treatment. This survey was repeated a year later to collect information on any new household members as well as changes in the past year.

We refer to the second type of survey as a “monthly survey.” Every month, participating households were visited by field workers who conducted interviews with all adults in the household. At this survey, we asked each person if they had had a malaria-like illness in the past month. If they did, we collected further information about the treatment steps they took for that illness (including whether they were tested and whether they took an ACT) as well as their beliefs about malaria testing and treatment. The household respondent provided this information for individuals under the age of 18.

The third type of survey is referred to as the “sick visit survey.” During the study period, household members were asked to contact the study team whenever they, or a child, felt unwell with suspected malaria. A trained field worker would visit the household to test the sick individual using an RDT. If the test result was positive, the participant was referred to the nearest pharmacy with a voucher to purchase a free Artemether Lumefantrine (AL) that was paid for by the study (this is the recommended first-line ACT for malaria in Kenya) [35]. If the individual tested negative, they were referred for further care at the nearest health facility. Severe cases were also referred for further management. During the visit, the individual (or the parent/guardian if the sick person was under age 18) was surveyed to ask their beliefs about the likelihood their illness was malaria (both immediately before the test and after receiving their result) as well as their confidence in malaria RDTs and ACTs.

### Statistical analysis

Confidence in malaria RDTs and in ACTs was assessed in all three surveys but on different subsets of the sample. In the annual surveys everyone aged 18 and above was asked questions about their confidence in malaria testing and treatment. In the monthly surveys, beliefs were elicited only for those who reported having a malaria illness in the past month. Lastly, in the sick visit surveys, people requesting and receiving an RDT from the study team were asked about confidence in testing and treatment.

We examined the relationship between 1) confidence in RDTs and the decision to be tested/adherence to the test result 2) the decision to test/test adherence and subsequent reported confidence in RDTs and ACTs 3) how individual beliefs changed over time in relation to cumulative experience with testing (long-term trend) and 4) how beliefs about an illness are updated in response to information from a test in real-time.

We used data from the most recent monthly survey or annual survey to examine how prior confidence in testing affected an individual’s decision to be tested for a malaria-like illness and also their adherence to the test result. We also used the monthly surveys to assess how adherence to an RDT result was associated with subsequent confidence in malaria RDTs and ACTs. The monthly surveys were also used to observe trends in ACT and RDT confidence over the study period.

To examine how experience with RDT testing affects people’s confidence in testing and treatment, we combined data from the annual surveys with the sick visit data. The annual surveys assessed individuals’ confidence in malaria RDTs and ACTs regardless of whether they were tested for malaria, and therefore we could analyze changes in these beliefs between the two annual surveys based on testing experience. We used the sick visit surveys to determine whether the individual was tested with an RDT from the study team between the two annual surveys and the number of times tested.

Lastly, we used data from the sick visit surveys to determine whether people used information from their RDT results to update their beliefs about the likelihood that their illness is malaria.

Beliefs about RDT accuracy were assessed using the question “If you have a fever and your malaria RDT is negative, how likely is it that the test is correct?” (with similar framing for a positive test result). For beliefs about ACT effectiveness, we asked “If you have malaria, and you take AL, how likely is it that you will be completely better in 3 days?” Lastly, for questions about the likelihood that their illness is malaria, we asked “How likely is it that the illness is malaria?” For all beliefs questions, responses were given on a 5-point Likert scale from “Very Likely” to “Very Unlikely.” As in other studies [21, 36], in order to simplify the analysis and interpretation of the results, we dichotomized these beliefs into a binary variable that consisted of “Very Likely” compared to all other responses. In the case of children under the age of 18, we used the responses of the household respondent. The household respondent was either the male or female head of the household and was generally the child’s parent or primary caregiver. We defined adherence to the test result as taking an ACT if they tested positive for malaria, and not taking an ACT if they tested negative for malaria.

We first conducted a descriptive analysis and present summary statistics on the sample in terms of proportions and means/medians. We then examined associations between beliefs and behavior using logistic regressions. We adjusted our standard errors for clustering of the outcome by household. We conducted both bi-variate analyses as well as adjusted regressions that included village fixed effects as well as controls for age, gender, education (less than primary versus primary or more), whether they slept under a bed net the previous night, whether they own more than an acre of land, and whether they got water from a protected source. The last two variables were included as measures of socio-economic status that varied within the sample. In most cases, we present and discuss the adjusted results in the text. All analyses were conducted using STATA 15.1 [37].

## Results

### Sample characteristics

The sample included 36 households, which consisted of 280 individuals (60 of whom were added over the course of the study period). The additional number of participants was a result of new members joining the cohort households (for example through birth). The median age of the household respondent, who provided information for children under the age of 18, was 42 years (IQR = 23) and 15 (42%) were female. 21 respondents (58%) had at least a primary education (Table 1, Panel A).

**Table 1:** Sample characteristics

**Panel A: Household Characteristics (*N* = 36)**	
	Median (IQR) or N(%)
Age of Household Respondent	41.5 (33.0, 56.0)
Household Respondent is Female	15 (41.7%)
Education Level of Household Respondent	
Less than primary	15 (41.7%)
Primary education or more	21 (58.3%)
Main source of drinking water	
Piped/protected source	26 (72.2%)
Unprotected source	10 (27.8%)
Owns more than one acre of land	16 (44.4%)
Household size	5.0 (4.0, 7.5)
**Panel B: Individuals (*N* = 280)**	
	N(%)
Female	151 (53.9%)
Adult 18 years or older:	110 (39.3%)
*Among Adults 18 years or older:*	
Education	
Less than a primary education	43 (39.1%)
Primary education or more	67 (60.9%)
Heard of RDTs	92 (87.6%)
Previously had an RDT (among those who have heard of RDTs)	85 (94.4%)
Beliefs about Malaria at Baseline	
Believe positive RDT very likely correct	82 (92.1%)
Believe negative RDT very likely correct	55 (62.5%)
Believe AL very effective in treating malaria	60 (60.0%)
Reported malarial illness over study period	227 (84.7%)
Number of study RDTs received	
0	56 (20.0%)
1	41 (14.6%)
2	27 (9.6%)
3 or more	156 (55.7%)
**Panel C: Monthly Surveys (*N* = 5617)**	
	N (%)
Reported malaria illness in past month	909 (16.2%)
Had RDT for malaria illness	638 (70.2%)
Tested positive for malaria	337 (52.8%)
Adhered to positive test result	323 (95.8%)
Adhered to negative test result	182 (60.7%)

The household respondent provided information on treatment of malarial illnesses for children under the age of 18. Their beliefs about RDTs and AL were also used for children under the age of 18

Of the 280 individuals in the sample (including the household respondent), 151(54%) were female, and 110 (39%) were aged 18 or above. Among the 110 individuals aged 18 or older, 67 (61%) had at least a primary level education. At the baseline survey, 92 (88%) had previously heard of RDTs, and among those who had, 85 (94%) also had previous experience with an RDT (Table 1, Panel B).

At baseline, confidence in RDTs and ACTs was low, in spite of high levels of awareness and experience with this technology. Among those individuals above the age of 18, 82(92%) believed that a positive RDT result was “very likely” to be correct, however only 55 (63%) believed that a negative RDT result was “very likely” to be correct. In addition, only 60 (60%) believed that AL was “very effective” in treating malaria (Table 1, Panel B).

We conducted 5,617 household surveys between June 2017 and December 2019 (including both monthly and annual surveys). In those surveys, 909 people (16%) reported having a malaria-like illness in the past month and 638 (70%) of those were tested with an RDT (from either the study team or elsewhere). 337 (53%) of those tests were reported as being positive for malaria. While 323 (96%) people adhered to a positive test result, only 182 out of the 300 (61%) who reported testing negative adhered to their test result (Table 1, Panel C).

### Confidence in malaria testing and treatment behavior

Table 2 shows the association between confidence in malaria RDTs and two key malaria treatment behaviors: whether an individual was tested with an RDT when they had a fever or malaria-like illness and whether an individual who was tested adhered to a negative RDT result. We focused on adherence to a negative test because adherence to a positive test was already very high. Compared to those with lower confidence in RDTs, those who believed a negative RDT was “very likely to be correct” were not more likely to get tested with an RDT (aOR = 1.31, 95% CI [0.866 1.976], *P* = 0.203), but, when they were tested with RDT, had 78% higher odds of adhering to a negative RDT result (aOR = 1.78, 95% CI [1.079 2.934], *P* = 0.024).

**Table 2:** Association between confidence in testing and treatment behavior

	**Tested with RDT**		**Adhered to negative RDT result**	
	(1)	(2)	(3)	(4)
*Coefficient on:*				
Believed Neg. RDT very likely correct prior to malaria illnesss	1.45 (0.29)	1.31 (0.28)	2.21** (0.54)	1.78* (0.45)
Mean of outcome in ref. group	0.67	0.67	0.48	0.48
Number of observations	863	863	295	295

Beliefs are those of the household respondent if the individual was under the age of 18. Results are from logistic regression models and coefficients are expressed in terms of odds ratios. Columns 1 and 3 are simple bi-variate regressions, while columns 2 and 4 include the following controls: age and gender of the individual, education level (of the respondent if the individual was under 18), whether the individual slept under a net the previous night, the main source of household drinking water, whether the household owns more than one acre of land and village fixed effects. *p < 0.05, **p < 0.01

### Experience with testing and confidence in testing and treatment

**Fig. 1:**
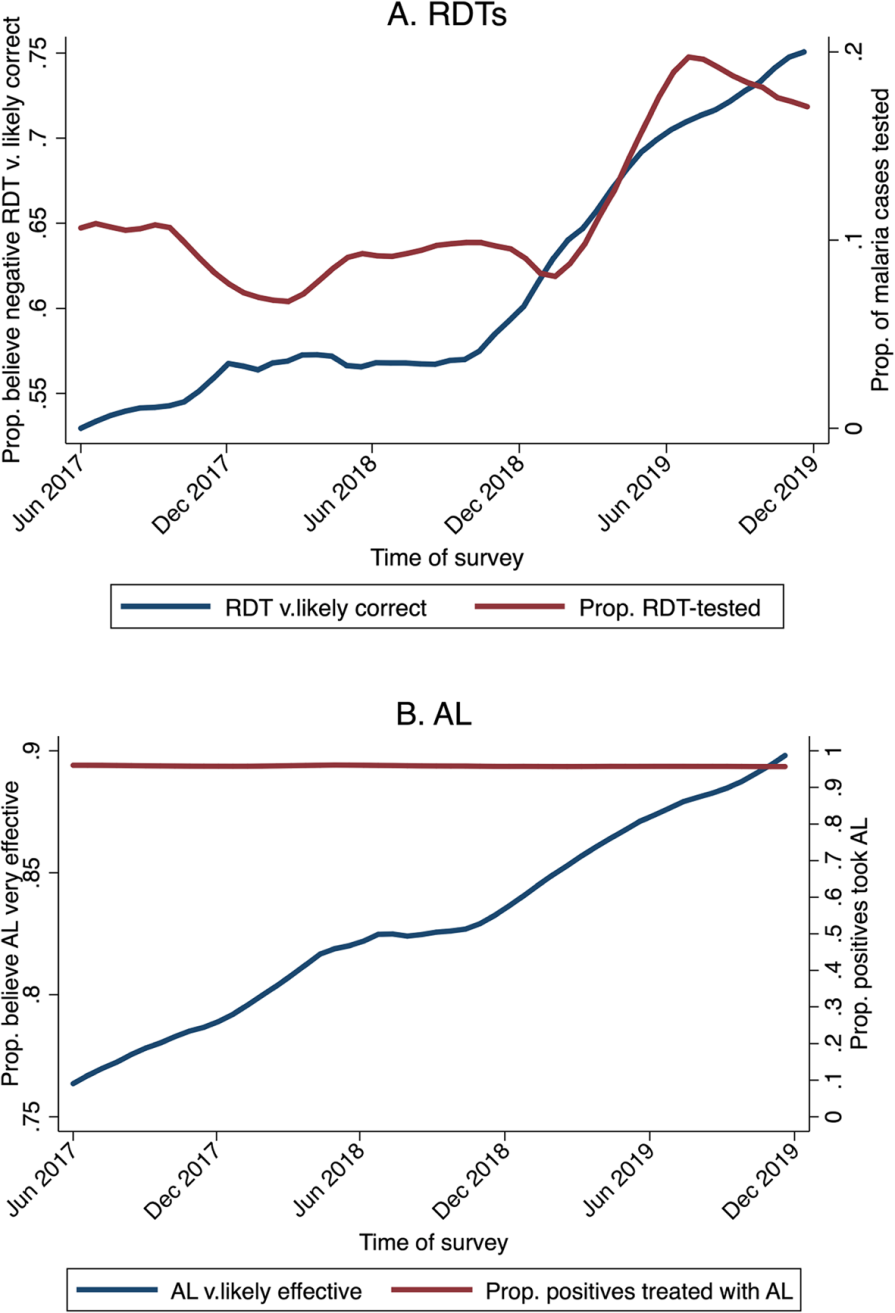
Confidence in RDTs (blue line, Panel A) and in AL (blue line, Panel B) over the survey period. Notes: Red lines indicate the proportion of illnesses tested with an RDT (Panel A) and the proportion of RDT-positives treated with AL (Panel B) over the same time period. Data is from monthly surveys and therefore only includes people who reported a malaria illness in the past month. Beliefs are those of the household respondent for children under 18

Over the 30-month study period, monthly beliefs data collected from all individuals who had a malaria-like illness, regardless of whether they were tested, show that confidence in both RDTs and ACTs increased steadily over time (Fig. 1). For RDTs, the proportion of people who said they believed a negative RDT was “very likely” to be correct increased from approximately 55% to 75%. The proportion of people who believed AL was “very likely” effective in treating malaria increased from approximately 75% to nearly 95%.

**Fig. 2:**
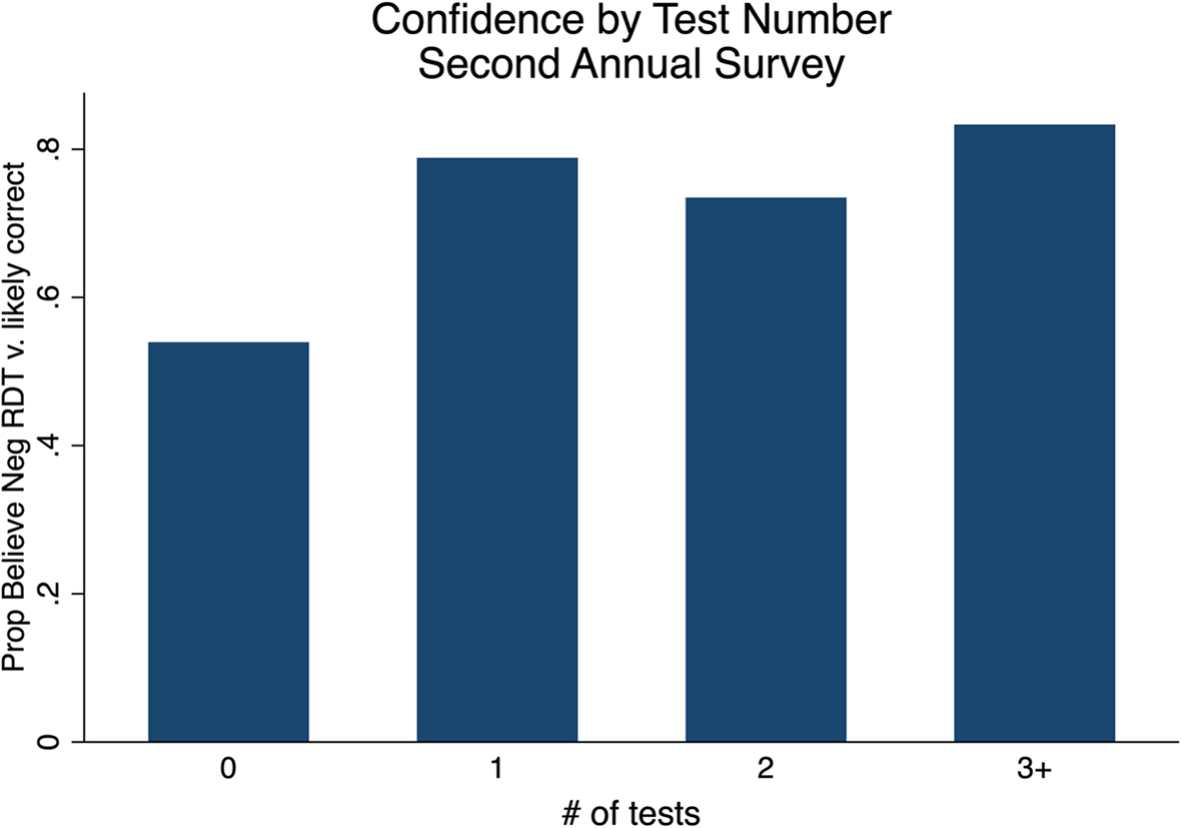
Confidence in RDTs by the number of tests an individual had over the first year of the study. Notes: Number of tests are limited to those that were performed by the study team. Beliefs are those of the respondent for children under 18. The differences between no RDTs and 1, 2, or 3 + RDT categories are statistically significant at *P* < 0.05, none of the other pairwise comparisons are statistically significant

When we compared those who were tested for malaria with those who were not tested, we find further evidence that testing experience was associated with higher confidence in RDTs. Those who had any study RDT over the first study year had approximately three times higher odds of believing a negative RDT was “very likely” to be correct at the end of the year, controlling for their beliefs at the start of the study period (aOR = 3.63, 95% CI [1.718 7.679], *P* = 0.001). We find no evidence, however, that the *number* of tests people had over this time period was associated with their confidence in RDTs (Fig. 2).

We also didn’t find strong evidence that the results of the test influenced changes in beliefs; those who received at least one positive RDT over the first year had slightly higher odds of strong confidence in RDTs at the end of the year but this association did not hold in the adjusted model and was not observed among those who had received at least one negative RDT during that time (Table 3).

**Table 3:** Association between testing experience and confidence in test

	**Outcome: Believed Negative RDT very likely correct at second annual survey**					
	(1)	(2)	(3)	(4)	(5)	(6)
*Coefficient on:*						
Any RDT	3.44** (1.40)	3.63** (1.39)				
Negative RDT result			1.86 (0.65)	2.10 (0.82)		
Positive RDT result					2.40* (1.07)	2.30 (1.12)
Believed Neg. RDT very likely correct at first annual survey	2.68 (1.96)	4.30 (3.92)	2.59 (1.91)	3.90 (3.50)	2.40 (1.82)	3.79(3.70)
Mean of outcome in ref. group	0.539	0.539	0.650	0.650	0.617	0.617
Number of observations	179	179	179	179	179	179

Beliefs are those of the household respondent if the individual was under the age of 18. Information on whether the individual was tested and the test result was based on sick visit surveys by the study team. Results are from logistic regression models and coefficients are expressed in terms of odds ratios. Columns1,3,5 are regressions that control only for baseline beliefs (measured at the first annual survey) while columns 2, 4, and 6 include the following controls: age and gender of the individual, education level (of the respondent if the individual was under 18), the main source of household drinking water, whether the household owns more than one acre of land and village fixed effects. *p < 0.05, **p < 0.01

Lastly, we did not find any statistically significant association between having been tested with an RDT and the odds of saying that AL was “very likely” to be effective in treating malaria at the end of the first year (Appendix Table A1).

### Treatment behavior and confidence in testing and treatment

In Table 4 we show how adherence to the test result was associated with individuals’ subsequent confidence in RDT testing, controlling for their confidence in RDTs before the illness. We find that those who adhered to their malaria test result had approximately twice the odds of saying that a hypothetical negative RDT was “very likely” to be correct after the illness compared to those who did not adhere to the test result (aOR = 2.20, 95% CI [1.661 2.904], *P* < 0.001). When we split this out by adherence to a positive versus a negative test result, we find little effect of adherence to a positive test result (aOR = 1.07, 95% CI [0.316 3.594], *P* = 0.918), but a significant difference in confidence from those who adhered to a negative test result relative to those who did not adhere (aOR = 2.09, 95% CI [1.403 3.116], *P* < 0.001).

**Table 4:** Association between adherence to test result and confidence in testing

	**Outcome: Believed Negative RDT very likely correct after illness**					
	(1)	(2)	(3)	(4)	(5)	(6)
*Coefficient on:*						
Adhered to RDT	2.17** (0.31)	2.20**(0.31)				
Adhered to Positive RDT			0.89 (0.56)	1.07 (0.66)		
Adhered to Negative RDT					2.07** (0.43)	2.09**(0.43)
Believed Neg. RDT very likely correct before illness	1.30 (0.22)	1.19 (0.20)	1.44 (0.33)	1.31 (0.31)	1.26 (0.39)	1.16 (0.40)
Mean of outcome in ref. group	0.48	0.48	0.71	0.71	0.46	0.4
Number of observations	619	619	324	324	295	295

Beliefs are those of the household head if the individual was under the age of 18. Results are from logistic regression models and coefficients are expressed in terms of odds ratios. Columns1,3,5 are regressions that control only for beliefs before the illness while columns 2, 4, and 6 include the following controls: age and gender of the individual, education level (of the respondent if the individual was under 18), whether the individual slept under a net the previous night, the main source of household drinking water, whether the household owns more than one acre of land and village fixed effects. *p < 0.05, **p < 0.01

We also find some evidence that those who adhered to a positive test result were more likely to subsequently say that AL was “very effective” in treating malaria compared to those who did not adhere, however our results are not statistically significant (Appendix Table A2).

### Updating beliefs with test result

**Fig. 3:**
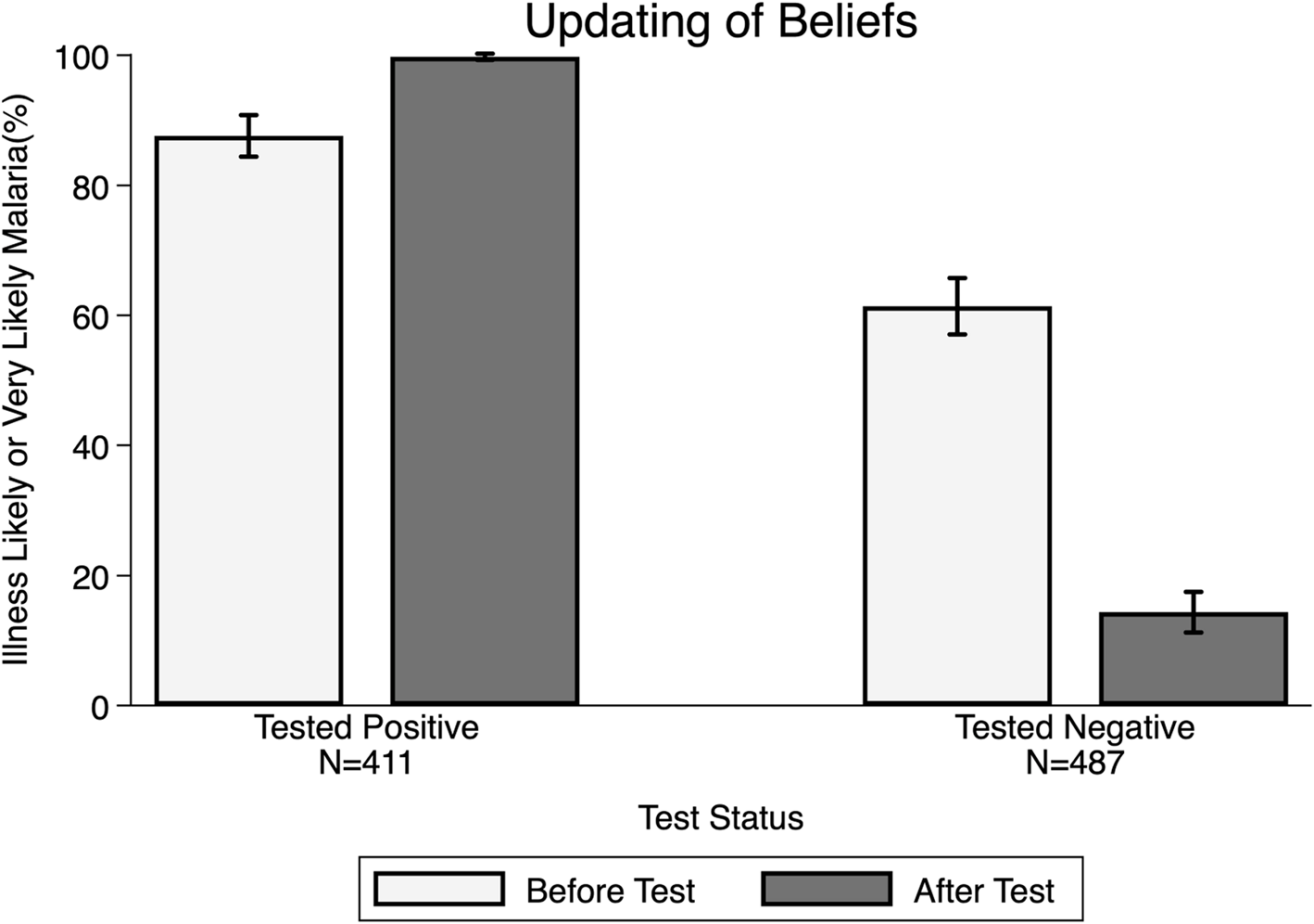
Beliefs about whether the illness is malaria before and after the test result. Data source is sick visit surveys. Beliefs are those of the household respondent for children under 18

Figure 3 demonstrates that individuals used the information from the test to update their beliefs about the likelihood that their (or their child’s) illness was malaria. For example, we find that for individuals who tested positive on their RDT, 87% said it was “very likely” their illness was malaria before the test, compared to nearly 100% after the test result (*P* < 0.001). For those who tested negative, 61% said it was “very likely” their illness was malaria before testing, compared to only 14% after the test result (*P* < 0.001).

We also found evidence that the degree to which people updated their beliefs based on the test results depended on their prior confidence in testing (Appendix Figure A1). Those who had said that a hypothetical negative RDT was “likely” or “very likely” to be correct, were more likely to revise downwards their belief that an illness was malaria after a negative test result, compared to those who said that they were less confident in a negative RDT result (*P* < 0.001).

## Discussion

This study found three main results. First, we showed that people’s beliefs about malaria testing affected their treatment behavior. In particular, we found that those who had higher confidence in RDT testing were more likely to adhere to a negative test result. We did not find that confidence in testing affected whether an individual got tested, similar to a previous study [38]. However, as in that study, the bar to testing was very low for participants: the RDT was free, a member of the study team would come and test at the household, and participants could get a free ACT if they tested positive for malaria. Our results differ from Maffioli et al. [36] however, in that they did not find that beliefs predicted adherence to the test result, possibly because individuals had less experience with RDTs at that time.

Second, we found evidence that treatment experiences also affected people’s subsequent beliefs about testing. For example, we showed that those who had an RDT during the first year of the study period had higher confidence in RDTs at the end of the year than those who were not tested during that time (controlling for their initial confidence in testing). In addition, we also showed that those who adhered to a negative test result were more likely to express high confidence in a hypothetical negative test at the next survey than those who did not adhere (once again controlling for their initial confidence in a negative test). These results suggest that people learn from their experience with testing and treatment. Our results are consistent with findings from population-level studies that show that greater access to testing increases people’s confidence in malaria testing [38] and can reduce inappropriate use of ACTs [22, 39]. Unlike a previous study [40] however, we did not find any statistically significant effects of testing experience on confidence in AL, perhaps because confidence in AL was higher compared to confidence in RDTs, and adherence to a positive RDT was close to 100%, thus limiting the scope for learning about AL effectiveness.

Lastly, we demonstrated that people used the information from the test to update their beliefs in the way that we would expect. Those who tested positive revised upwards the likelihood that their illness was malaria, while those who tested negative revised downward their beliefs about the likelihood that their illness was malaria. This suggests that the information from malaria testing could play an important role in people’s treatment decisions.

There are several limitations in this study. Given that our study was conducted with 36 households in Western Kenya, it is possible that prevailing pre-conceptions among that group may not translate widely to other settings. Moreover, when considering individual-level beliefs, we do not account for the fact that household members could also learn from each other’s testing and treatment decisions (for example, a parent/guardian could learn from their own test results but also from those of their children). We did adjust our standard errors for clustering by household to account for the fact that observations within households were not independent from each other. Third, even though we controlled for initial beliefs as well as other demographic factors, it is likely that those who chose to be tested with an RDT were different from those who did not in unobservable ways. Lastly, the way people’s beliefs about malaria likelihood and beliefs about AL effectiveness were dichotomized (“very likely” compared to all others) means that the analysis focused on the degree to which respondents were fully confident in their response or had some uncertainty.

Nonetheless, our study design also has several strengths. Since we followed the same people over time, we could see how individual beliefs changed because of testing and treatment decisions, rather than focusing simply on population-level changes as testing becomes more available. Furthermore, we collected beliefs at multiple time points: before testing, after testing, and after treatment. This allowed us to observe how beliefs change at each treatment step.

## Conclusions

Overall, our results suggest that people’s beliefs have an important role in treatment behavior but also that treatment behavior can in turn influence those beliefs. In terms of policy implications, our results suggest that lowering the barriers to testing would not only increase access to malaria RDTs thus potentially improving appropriate use of ACTs, but could also be beneficial in terms of community learning about the value of these new treatment technologies. Strategies for increasing uptake of RDTs (and other new health technologies) could include large subsidies [41–43], and making them more convenient and accessible such as at local drug shops, [44, 45] or through community health workers.[22, 46, 47]. With lower barriers, people can experiment with the technology thereby gaining confidence in its value, and promoting further uptake and appropriate use. These strategies could also potentially be used for increasing confidence and uptake of similar health tools for other diseases such as COVID-19 and HIV.

## Supplementary Information

**Additional file 1: Appendix Table A1.** Association between Testing Experience and Confidence in AL. **Appendix Table A2.** Association between Adherence to Test Result and Confidence in AL. **Appendix Figure A1.** Change in beliefs illness likely malaria before and after negative test result by confidence in the test. Data source is sick visit surveys. Beliefs are those of the respondent for children under 18.

## Data Availability

The data is currently still in use by colleagues who are writing up on other aspects of the main study. However, these datasets used and/or analyzed during the current study can be availed by the corresponding author upon reasonable request.

## Abbreviations

ACT: Artemisinin-Combination Therapies
AL: Artemether Lumefantrine
HDSS: Health and Demographic Surveillance Site
RDT: Rapid Diagnostic Test
