# Supplementary material for: Experience and confidence in health technologies: evidence from malaria testing and treatment in Western Kenya
Source: BMC Public Health. 2022 Sep 6;22:1689. doi: 10.1186/s12889-022-14102-y (PMC9446607; doi:10.1186/s12889-022-14102-y)
Supplement: Supplementary file 1 — Additional file 1: Appendix Table A1. Association between Testing Experience and Confidence in AL. Appendix Table A2. Association between Adherence to Test Result and Confidence in AL. Appendix Figure A1. Change in beliefs illness likely malaria before and after negative test result by confidence in the test. Data source is sick visit surveys. Beliefs are those of the respondent for children under 18. [file 12889_2022_14102_MOESM1_ESM.docx]

**Supplementary Materials**

**Experience and confidence in health technologies: evidence from malaria testing and treatment in Western Kenya.**

**Appendix Figures and Tables**

**Appendix Table A1: Association between Testing Experience and Confidence in AL**

Notes: Beliefs are those of the household head if the individual was under the age of 18. Information on whether the individual was tested and the test result is based on sick visit surveys by the study team. Results are from logistic regression models and are expressed in terms of odds ratios. Columns 2, 4, and 6 include the following controls: age and gender of the individual, education level (of the respondent if the individual was under 18), the main source of household drinking water, whether the household owns more than one acre of land and village fixed effects. *p<0.05, **p<0.01

**Appendix Table A2: Association between Adherence to Test Result and Confidence in AL**

Notes: Beliefs are those of the household head if the individual was under the age of 18. Results are from logistic regression models and are expressed in terms of odds ratios. Columns 2, 4 and 6 include the following controls: age and gender of the individual, education level (of the respondent if the individual was under 18), whether the individual slept under a net the previous night, the main source of household drinking water, whether the household owns more than one acre of land and village fixed effects. *p<0.05, **p<0.01

**
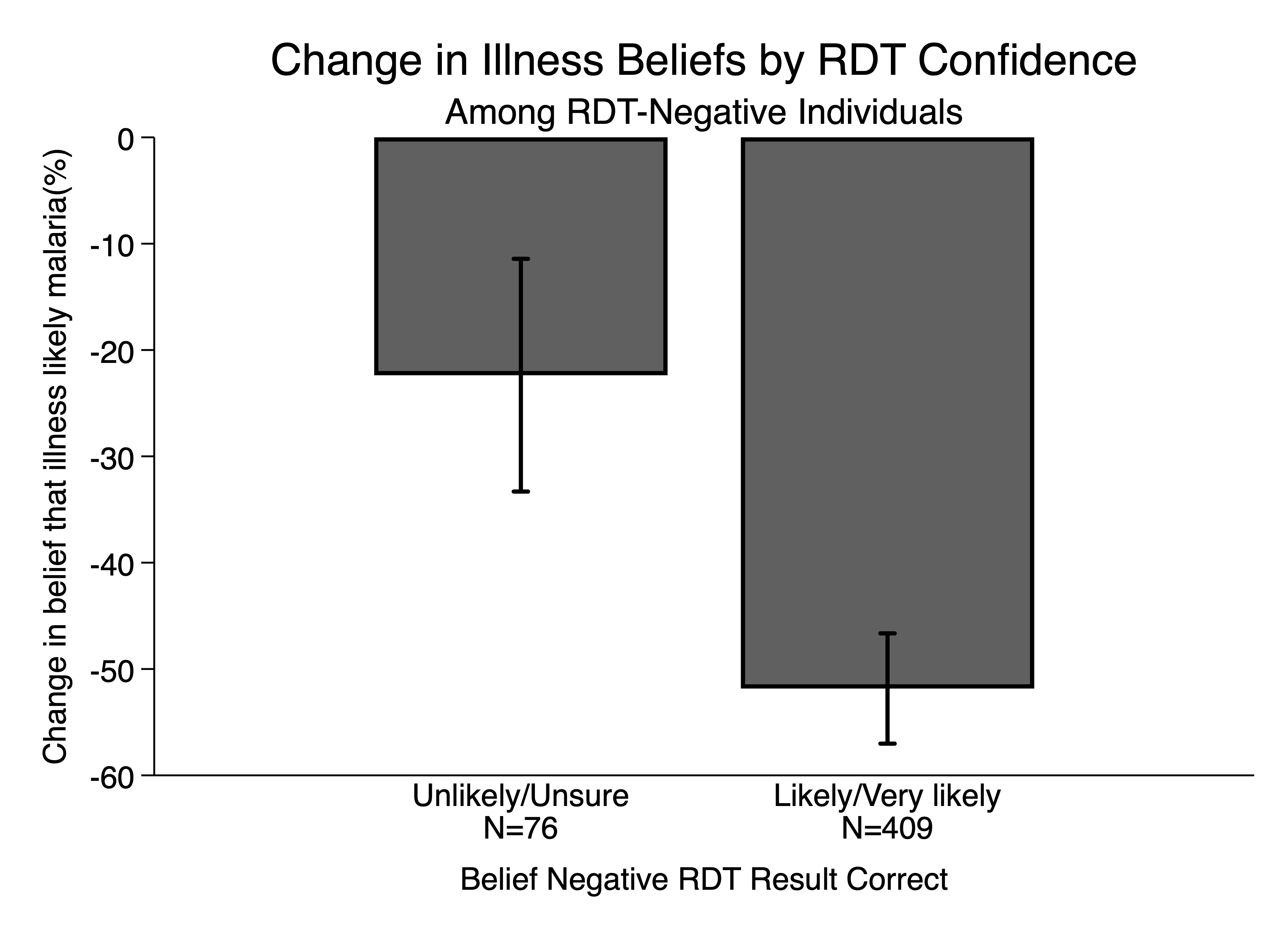
**

**Appendix Figure A1:** Change in beliefs illness likely malaria before and after negative test result by confidence in the test. Data source is sick visit surveys. Beliefs are those of the respondent for children under 18.
